# Kinetic Study of the Diels–Alder Reaction between Maleimide and Furan-Containing Polystyrene Using Infrared Spectroscopy

**DOI:** 10.3390/polym16030441

**Published:** 2024-02-05

**Authors:** Tongtong Wang, Dali Gao, Hua Yin, Jiawei Zhao, Xingguo Wang, Hui Niu

**Affiliations:** 1 Department of Polymer Science and Engineering, School of Chemical Engineering, Dalian University of Technology, Dalian 116024, China; 2 SINOPEC (Beijing) Research Institute of Chemical Industry Co., Ltd., Beijing 100013, China

**Keywords:** Diels–Alder reaction, kinetics, infrared spectroscopy

## Abstract

The Diels–Alder (D–A) reaction between furan and maleimide is a thermally reversible reaction that has become a vital chemical technique for designing polymer structures and functions. The kinetics of this reaction, particularly in polymer bulk states, have significant practical implications. In this study, we investigated the feasibility of utilizing infrared spectroscopy to measure the D–A reaction kinetics in bulk-state polymer. Specifically, we synthesized furan-functionalized polystyrene and added a maleimide small-molecule compound to form a D–A adduct. The intensity of the characteristic absorption peak of the D–A adduct was quantitatively measured by infrared spectroscopy, and the dependence of conversion of the D–A reaction on time was obtained at different temperatures. Subsequently, the D–A reaction apparent kinetic coefficient *k*_app_ and the Arrhenius activation energy *E*_a,D–A_ were calculated. These results were compared with those determined from ^1^H-NMR in the polymer solution states.

## 1. Introduction

The Diels–Alder (D–A) reaction, also known as the [4 + 2] cycloaddition reaction, was first proposed by Otto Diels and Kurt Alder. This reaction, which belongs to the click chemical reaction system [1], is not only a general method for the synthesizing of six-membered rings [2,3], but also one of the most extensively studied thermal reversible equilibrium reactions. The D–A reaction is a one-step synergetic reaction with a reversible process, and due to the absence of ionic reaction, it exhibits high adaptability to the reaction environment. The reaction produces a D–A cycloaddition product at low temperature, while a *retro-*Diels–Alder (*r*D–A) reaction occurs at high temperature, leading to the conversion of D–A cycloaddition back into the initial reactants [4]. Furan/maleimide (and its derivatives) is the most widely studied thermoreversible D–A reaction system [5,6]. The efficient construction of macromolecules based on the D–A reaction between furan and maleimide has become an interesting research field [7,8,9,10,11,12]. For instance, researchers can conveniently construct polymers with self-healing functions [13,14,15,16,17,18] or recycle thermosetting materials [19] by employing the D–A reaction. Furthermore, bifuran and bismaleimide can be utilized as monomers to fabricate polymers with complex structures directly through stepwise polymerization reaction [20,21]. However, due to the complexity of the reactions between macromolecules, it is crucial to study the kinetics of the D–A reaction in polymer systems.

Currently, the kinetics of the D–A reaction are studied using a range of methods including UV spectroscopy [17,22,23], ^1^H-NMR spectroscopy [24], rheological testing [25,26], and a combination of various techniques [27,28]. Gandini et al. [22] used UV spectroscopy to investigate the reaction kinetics of furfuryl acetate and *N*-methylmaleimide. The conversion of the D–A reaction at a certain time was determined by monitoring the variation in the conjugate characteristic peak of C=C-C=O in maleimide at 300 nm, and the Arrhenius activation energy *E*_a_ was reported as 40 ± 3 kJ mol^−1^. Wang et al. [24] utilized in situ ^1^H-NMR spectroscopy to study the D–A and *r*D–A reaction kinetics in linear polyurethanes synthesized by the D–A reaction. By analyzing the variation in the D–A characteristic peak intensity with temperature, the authors confirmed that the D–A reaction followed second-order kinetic law and determined the *E*_a_ to be 47 kJ mol^−1^. Polgar et al. [25] investigated the D–A reversible reaction in ethylene–propylene rubber using rheological testing. The researchers deduced that the D–A reaction followed second-order kinetics, while the *r*D–A reaction followed first-order kinetics by measuring the change in polymer modulus with time. The *E*_a,D–A_ was found to be 7.04 kJ mol^−1^ at 60~80 °C, and the *E*_a,*r*D–A_ was 57.9 kJ mol^−1^ at 130~140 °C. Additionally, the change in enthalpy and the change in entropy of the reaction were determined. In previous work, we synthesized thermoreversibly crosslinked ethylene–propylene rubber based on the D–A reaction and investigated its kinetics using rheological testing [29,30,31]. It was revealed that the structure of maleimide, as well as the molar ratio of maleimide to furan, influenced the *E*_a_ and the apparent kinetic coefficient *k*. By adjusting the furan/maleimide ratio, the *E*_a_ value was decreased from 51.78 kJ mol^−1^ to 19.29 kJ mol^−1^. These studies have significantly contributed to our understanding of the D–A reaction in polymer systems.

However, there exist practical obstacles when utilizing the aforementioned techniques to examine kinetics in polymer systems. For instance, UV and ^1^H-NMR spectroscopies are not ideal for detecting polymers that lack appropriate solvents across a broad temperature range, such as polyethylene and isotactic polypropylene. Furthermore, rheological methods are not suitable for analyzing D–A reactions in highly crystalline polymers or those with high glass transition temperatures, as rheological tests necessitate the polymers to remain in viscoelastic states. In addition, since polymers are predominantly used in bulk (i.e., without solvent), the studies of the D–A reaction in polymer bulk states are more important. Infrared spectroscopy is a classical analytical tool that is highly suited to bulk polymer analysis. Many studies qualitatively demonstrated the reversibility of the D–A reaction using this technique [32,33,34,35]. For instance, Singha et al. [36] employed FTIR to investigate the D–A reaction in the homopolymers of furfuryl methacrylate, finding that the *r*D–A reaction followed the first-order kinetic law with the *E*_a,*r*D–A_ of 54.25 kJ mol^−1^. Bose et al. [37] employed a combination of FTIR and rheological analyses to study the kinetics of the *r*D–A reaction in a one-system D–A polymer, which combined furan and maleimide groups with a polymethacrylate copolymer. They determined the *E*_a_ of the *r*D–A reaction to be 64.8 kJ mol^−1^.

The kinetics of the *r*D–A reaction reflect the material’s reprocessability, while the kinetics of the D–A reaction reflect the recoverability of the material properties. Notably, the D–A reaction in polymers is affected not only by temperature, but also by the structure and mobility of the reactant groups. In this paper, we demonstrate that infrared spectroscopy can be used for the quantitative calculation of the D–A reaction conversion and *E*_a_, and the accuracy of the results is verified using ^1^H-NMR spectroscopy. 

## 2. Experiment

### 2.1. Materials

Styrene (St, 99%), *N*-phenylmaleimide (99%), 1-dodecyl-1H-pyrrole-2,5-dione (95%), and 2,2′-Azobisisobutyronitrile (AIBN) were purchased from Energy Chemical, Beijing, China. The styrene was washed with 10% aqueous NaOH, dried over magnesium sulfate, and then fractionally distilled under reduced pressure.

### 2.2. Synthesis of Furan-Functionalized Polystyrene (PSF)

**Scheme 1:**
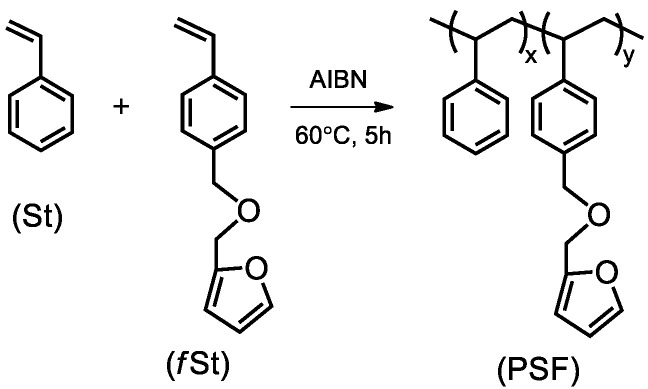
Synthesis route of furan-functionalized polystyrene PFS.

Furan-functionalized styrene (*f*St) was synthesized by reference to the literature method [38]. The poly (St-co-*f*St), PSF, was prepared via free radical polymerization. The synthesis route of PSF is shown in Scheme 1. In a typical process, 61.7 mmol *f*St, 144.0 mmol styrene (St), and 0.56 g initiator AIBN were added to a flask under the protection of a nitrogen atmosphere, and the product was collected after polymerization at 60 °C for 5 h. The produced polymer was dissolved in dichloromethane and precipitated in methanol. The resulting precipitate was washed using dichloromethane to remove the unreacted monomers and initiators. The product was filtered and vacuum-dried at 40 °C before being characterized by ^1^H-NMR spectroscopy (Figure S1).

### 2.3. Diels–Alder Reaction of PSF with Maleimides

The cycloaddition product of PSF with *N*-phenylmaleimide (Ma), PSF-Ma, formed by the D–A reaction was synthesized as follows: PSF and Ma (with the molar ratio of furan/maleimide = 1/2) were dissolved in dichloromethane, and then the solution was heated under reflux in a nitrogen atmosphere for 24 h at 40 °C. The resulting product was precipitated in methanol, and the unreacted monomers were washed using dichloromethane and dried under vacuum at 40 °C. The structural analysis showed that 25.0% of the furyl group in the PSF underwent the D–A reaction.

The cycloaddition product of PSF with 1-dodecyl-1H-pyrrole-2,5-dione (Mb), PSF-Mb, was prepared as follows: PSF and Mb (with the molar ratio of furan/maleimide = 1/2) were dissolved in chloroform, and the solution was heated under reflux in a nitrogen atmosphere for 24 h at 60 °C. The product was precipitated in methanol and the unreacted monomers were washed away with hexane, and finally dried under vacuum at 40 °C. The structural analysis showed that 19.7% of the furyl group in the PSF underwent the D–A reaction.

### 2.4. Characterization

Nuclear magnetic resonance spectra (^1^H-NMR) were recorded on a Vaian DLG 400 MHz spectrometer (Vaian, CA, USA) using C_2_D_2_Cl_4_. Fourier-transform infrared spectroscopy (FTIR) spectra were obtained using a Nicolet 6700 FTIR spectrometer (ThermoFisher, Waltham, MA, USA), and the transmission mode with 32 scans was applied.

## 3. Results and Discussion

**Figure 1:**
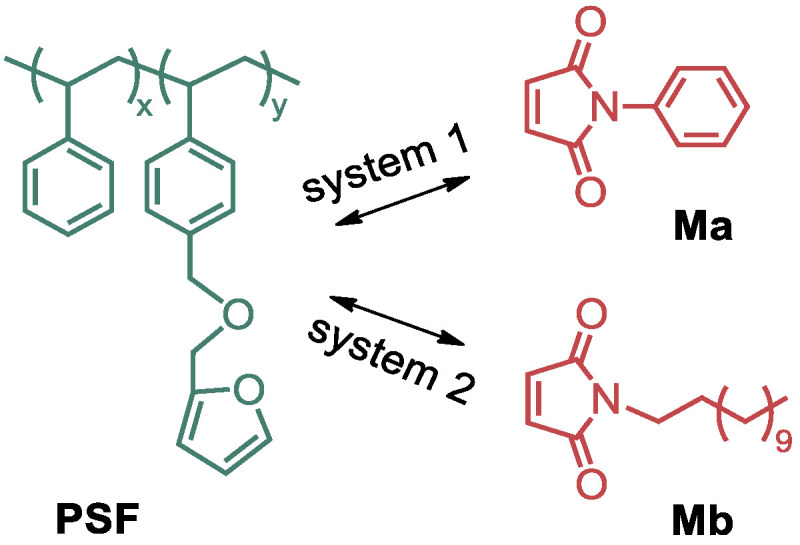
Furan/maleimide systems designed in this work.

Two furan/maleimide systems were designed to investigate the kinetics of the thermoreversible D–A reaction. The furan moiety was incorporated into polystyrene (PSF) through the copolymerization of styrene (70 mol%) and furyl-functionalized styrene (30 mol%). The maleimide moiety was a small molecule with either aromatic or aliphatic substituent groups, denoted as Ma and Mb, respectively, as illustrated in Figure 1.

### 3.1. Diels–Alder Reaction between PSF and Ma

**Scheme 2:**
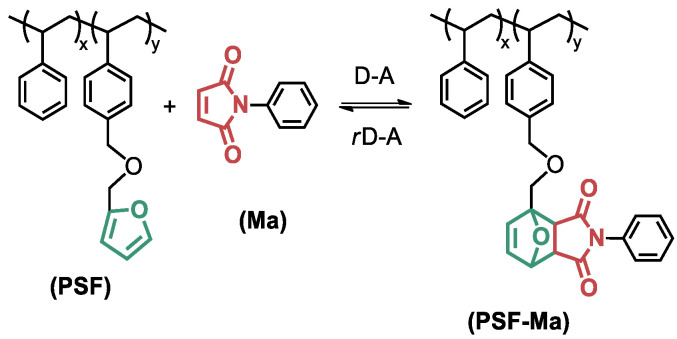
Reversible Diels–Alder reaction between PSF and Ma.

**Figure 2:**
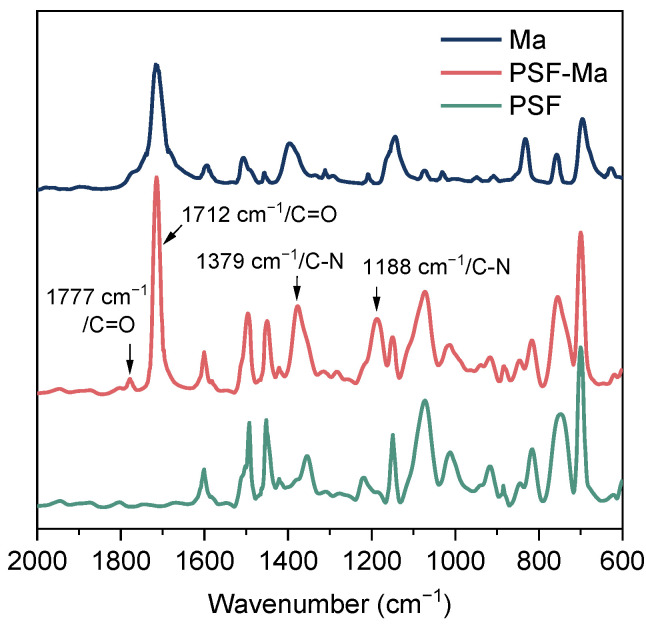
FTIR spectra of PSF, Ma, and PSF-Ma.

The reversible D–A reaction between PSF and Ma is presented in Scheme 2. The formation of the D–A cycloaddition product PSF-Ma was identified by FTIR. Figure 2 compares the FTIR spectra of PSF, Ma, and PSF-Ma. The characteristic absorption peaks at 1777, 1712, 1379, and 1188 cm^−1^ [32,37], which emerge or increase in intensity, are representative of the PSF-Ma adduct.

**Figure 3:**
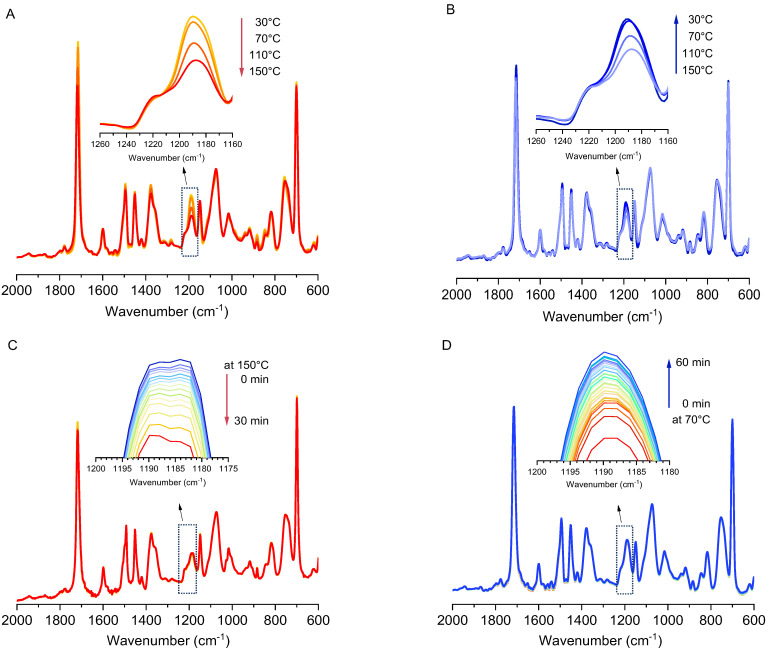
FTIR spectra of (**A**) the heating procedure of PSF-Ma from 30 °C to 150 °C, (**B**) the cooling procedure of PSF-Ma from 150 °C to 30 °C, (**C**) holding the temperature at 150 °C for 30 min, and (**D**) holding the temperature at 70 °C for 60 min.

By gradually increasing the temperature of the infrared spectroscopy pool from 30 °C to 150 °C, and observing the FTIR spectra of PSF-Ma, as depicted in Figure 3A, it was discovered that the intensity of the C-N vibration peak at 1188 cm^−1^ steadily decreased, while cooling the sample pool from 150 °C to 30 °C resulted in an increase in the intensity of this peak, as shown in Figure 3B. This indicates that the D–A/*r*D–A reaction in the bulk state of the PSF/Ma system can be monitored by measuring the absorption at 1188 cm^−1^. The D–A cycloaddition product PSF-Ma was heated to 150 °C, and an infrared spectrum was obtained every 2 min to observe the changes in the sample structure over time, as shown in Figure 3C. It was observed that the peak at 1188 cm^−1^ gradually weakened with time at 150 °C, indicating the gradual dissociation of D–A cycloaddition in PSF-Ma via the *r*D–A reaction occurring at high temperature. Furthermore, when the dissociated PSF/Ma was rapidly cooled from 150 °C to 70 °C to induce the D–A cycloaddition reaction, as illustrated in Figure 3D, the gradual increase in absorption at 1188 cm^−1^, with a detection interval of 2 min, demonstrated the feasibility of determining the D–A conversion variation at a specific time in the PSF-Ma bulk state using FTIR.

The kinetics of the D–A reaction were investigated by monitoring the FTIR absorbance at 1188 cm^−1^ at different temperatures. Initially, it is assumed that the D–A equilibrium is dominated by the D–A cycloaddition reaction at low temperatures and by the dissociation reaction of the D–A adduct at high temperatures, as shown in Scheme 2. For kinetic studies, a simplified n-level reaction model is often used to derive the corresponding kinetics equations (Equation (1)) [25], where *k*_app_ is the apparent kinetic coefficient, and x is the reaction conversion at time t. When the reaction kinetic order n is 1, 2, and 3, Equation (1) can be converted to Equations (2)–(4), respectively.

(1) $$\frac{dx}{dt}=k_{app}(1-x{)}^{n}$$

(2) $$\ln\frac{1}{1-x}=k_{1}t(n=1)$$

(3) $$\frac{1}{1-x}=k_{2}t(n=2)$$

(4) $$\left(\frac{1}{1-x}{)}^{2}=k_{3}t(n=3\right)$$

**Figure 4:**
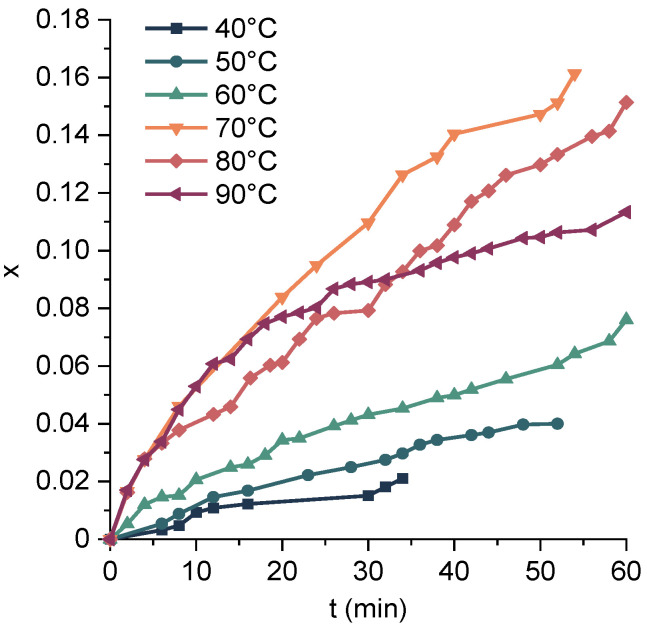
Dependence of conversion x on reaction time in PSF/Ma system derived from FTIR results at 40 °C, 50 °C, 60 °C, 70 °C, 80 °C, and 90 °C, respectively.

In this work, the D–A reaction conversion x was determined by absorbance at 1188 cm^−1^ from Equation (5):

(5) $$x=\frac{A_{t}-A_{0}}{A_{30\;{}^{\circ}C}-A_{150\;{}^{\circ}C,30\min}}$$

where A_t_ and A_0_ are the absorbance value at the designed temperature at time t = t and t = 0, respectively; and A_30 °C_ and A_150 °C, 30min_ are the absorbance at 30 °C (the end of the D–A reaction) and the absorbance at 150 °C after 30 min (the end of the *r*D–A reaction). The following steps were adopted: (1) keeping the D–A cycloaddition product PSF-Ma at 150 °C for 30 min to ensure that the *r*D–A reaction had totally completed; (2) rapidly cooling the infrared spectroscopy sample pool to the designed temperature; and (3) reading the absorbance at 1188 cm^−1^ at different times to calculate the D–A reaction conversion x according to Equation (5). The D–A reaction between the PSF and Ma was performed at six different temperatures, namely, 40 °C, 50 °C, 60 °C, 70 °C, 80 °C, and 90 °C. The absorbance was recorded with time and is presented in Figure S2. Using Equation (5), the relationship between x and time at different temperatures in the D–A reaction is plotted in Figure 4. It is evident that x increased almost linearly with time, and the slope of the curves increased with the temperature until it reached its maximum at 70 °C. Further increasing the temperature to 80 °C and 90 °C resulted in a decline in the slope, indicating that the *r*D–A reaction could not be ignored at these temperatures.

**Figure 5:**
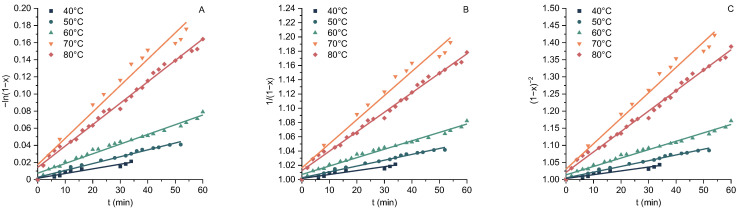
Fitting the conversion data to (**A**) first-order, (**B**) second-order, and (**C**) third-order reaction kinetics to determine the apparent kinetic coefficient *k*_app_ of D–A reaction between PSF and Ma at 40 °C, 50 °C, 60 °C, 70 °C, and 80 °C based on the FTIR results.

**Figure 6:**
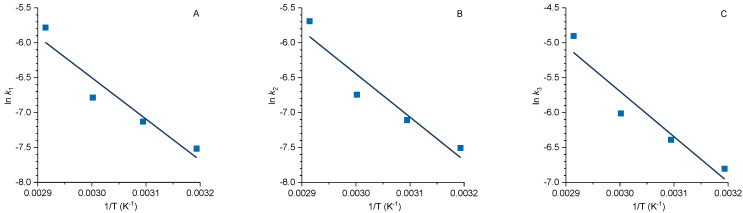
Fitting the Arrhenius plot to (**A**) first-order, (**B**) second-order, and (**C**) third-order reaction to determine the *E*_a,D–A_ and *k*_0,D–A_ of the D–A reaction between PSF and Ma based on the FTIR results.

At a certain temperature, the apparent kinetic coefficient *k*_app_ of the D–A reaction was determined from the conversion data by fitting the x to Equations (2), (3), and (4), respectively, and the resulting slope was *k*_1_, *k*_2_, *k*_3_, as shown in Figure 5. The *k*_app_ values are summarized in Table 1, and the linear fits were found to be satisfactory, with *R*^2^ values close to 1. To determine the Arrhenius activation energy, *E*_a,D–A_, of the D–A reaction, the *k*_app_ values at different temperatures were used to construct Arrhenius plots, as shown in Figure 6. The slope of the plot corresponds to *E*_a,D–A_, while the intercept corresponds to the pre-exponential factor *k*_0,D–A_, as defined by Equation (6).

(6) $$\ln k_{app}=-\frac{E_{a,D-A}}{RT}+\ln k_{0,D-A}$$

All of the first-, second-, and third-order coefficients (*k*_1_, *k*_2_, *k*_3_) increased with increasing temperature from 40 °C to 70 °C, but decreased at 80 °C. This trend suggested that heating promoted the rapid completion of the D–A reaction within the temperature range of 40~70 °C, whereas at 80 °C, the reaction was influenced by the *r*D–A process. The *E*_a,D–A_ was calculated based on the data obtained at 40 °C, 50 °C, 60 °C, and 70 °C, and the resulting value of *E*_a,D–A_ was approximately 50 kJ mol^−1^, as shown in Table 1. It was observed that both second-order and third-order kinetics fit the entire temperature range. To further determine the kinetics reaction order, additional studies utilizing ^1^H-NMR spectroscopy will be conducted.

**Figure 7:**
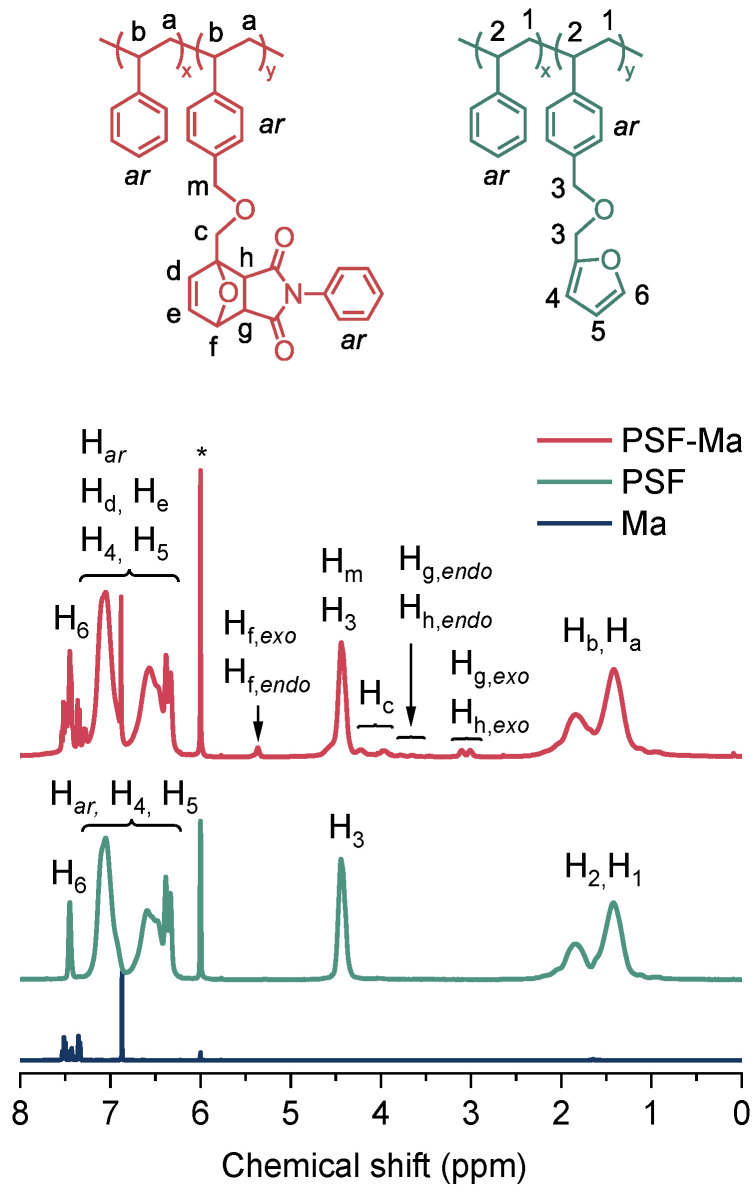
^1^H-NMR spectra of PSF, Ma, and PSF-Ma. (* solvent: C_2_D_2_Cl_4_).

PSF-Ma was characterized using ^1^H-NMR spectroscopy. Figure 7 presents the assignments for the various resonances, including those that distinguish between *endo* and *exo* D–A stereoisomers. Compared to PSF, PSF-Ma exhibited characteristic D–A peaks at 5.37 ppm, 3.96~4.23 ppm, 3.65~3.78 ppm, and 3.01~3.11 ppm. The composition of PSF-Ma can be determined by analyzing the characteristic peak integral at 3.96~4.23 ppm, combined with the integral at 3.96~4.44 ppm. The results indicate that 25.0 mol% of the furyl group in PSF underwent the D–A reaction, and the F/M ratio (molar ratio of furan and maleimide) in the PSF-Ma was 4:1.

**Figure 8:**
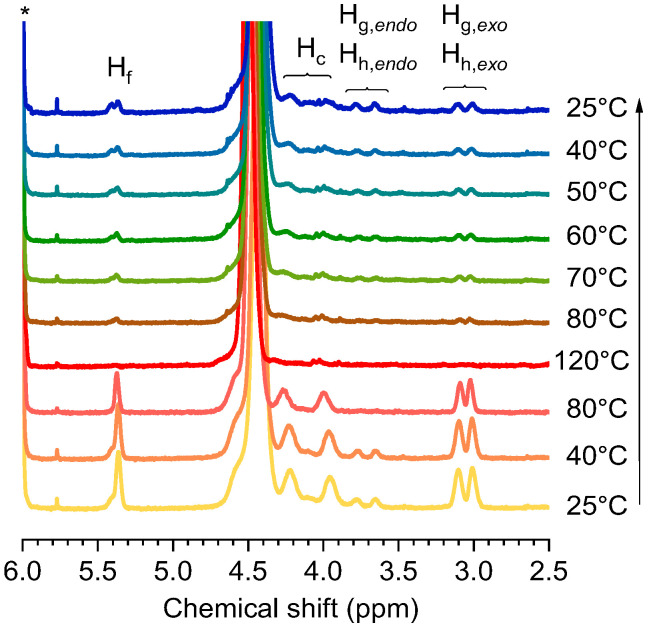
^1^H-NMR spectra of PSF-Ma at variable temperatures. (* solvent: C_2_D_2_Cl_4_).

In order to investigate the kinetics of the D–A reaction, the in situ ^1^H-NMR spectroscopy was utilized, which further validated the precision of the FTIR results. A temperature cycle was performed, ranging from 25 °C to 120 °C, to obtain a variable temperature ^1^H-NMR spectrum, as depicted in Figure 8. The reaction between PSF and Ma in the PSF-Ma was carried out in C_2_D_2_Cl_4_ (under a nitrogen atmosphere) and characterized using real-time ^1^H-NMR spectroscopy. The analysis of the ^1^H-NMR spectra of the PSF-Ma revealed a continuous decrease in the D–A characteristic peaks at 5.37 ppm, 3.96~4.23 ppm, 3.65~3.78 ppm, and 3.01~3.11 ppm, while the characteristic peak of D–A at 4.44 ppm increased during the heating process, indicating the occurrence of the *r*D–A reaction at high temperatures. Conversely, during the cooling process, the characteristic peaks exhibited an opposite trend, indicating the occurrence of the D–A reaction at low temperatures.

**Figure 9:**
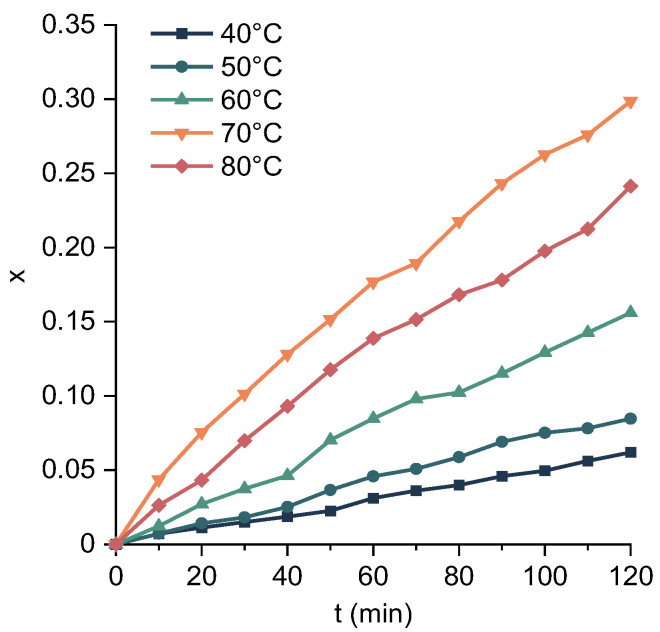
Dependence of conversion x on reaction time in PSF/Ma system derived from ^1^H-NMR results at 40 °C, 50 °C, 60 °C, 70 °C, and 80 °C, respectively.

The PSF-Ma adduct was subjected to the *r*D–A reaction at 120 °C, followed by cooling to various temperatures (40 °C, 50 °C, 60 °C, 70 °C, and 80 °C) for 2 h in C_2_D_2_Cl_4_, as shown in Figure S3. The reaction was carried out in a ^1^H-NMR tube under a nitrogen atmosphere and monitored in real time using ^1^H-NMR spectroscopy. One spectrum was scanned every 10 min. The results showed that the characteristic peak of D–A adduct completely disappeared after 20 min at 120 °C, indicating the occurrence of the *r*D–A reaction at high temperature. As the temperature was gradually lowered to below 80 °C, the characteristic peak of D–A adducts steadily increased with the extension of time, indicating the occurrence of the D–A reaction at low temperature. The feasibility of calculating the variation in the conversion x with time using in situ ^1^H-NMR was confirmed. The D–A reaction conversion x was determined from Equation (7):

(7) $$x=\frac{2A_{c}}{A_{3}+A_{m}+A_{c}}=\frac{2(A_{g}+A_{h})}{A_{3}+A_{m}+A_{c}}$$

where A_3_, A_c_, A_m_, A_g_, and A_h_ denote the integrated areas of the peaks 3, c, m, g, and h, respectively. The relationship between x and time at each temperature is illustrated in Figure 9. Within the temperature range of 40~70 °C, an increase in temperature corresponds to an accelerated conversion at a constant reaction time. However, a decline in x is evident at temperatures exceeding 80 °C, signifying the commencement of the *r*D–A reaction. Accordingly, it is inferred that the optimal temperature for the D–A reaction is 70 °C, where the conversion attains its maximum.

**Figure 10:**
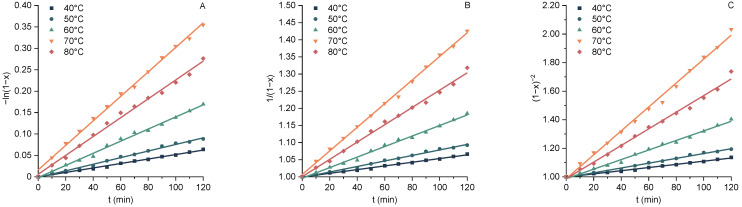
Fitting the conversion data to (**A**) first-order, (**B**) second-order, and (**C**) third-order reaction kinetics to determine the apparent kinetic coefficient *k*_app_ of D–A reaction between PSF and Ma at 40 °C, 50 °C, 60 °C, 70 °C, and 80 °C based on the ^1^H-NMR results.

The kinetics of the D–A reaction during the initial 120 min were simulated using ^1^H-NMR analysis, as illustrated in Figure 10. The first-, second-, and third-order coefficients (*k*_1_, *k*_2_, *k*_3_) were calculated and are listed in Table 1. It was observed that the values of *k*_1_, *k*_2_, and *k*_3_ increased as the temperature increased from 40 °C to 70 °C, but decreased at 80 °C. This suggests that a moderate increase in temperature within the range of 40~70 °C is beneficial for promoting the rapid completion of the D–A reaction. Comparing the results of three series, it was found that the linear fitting value *R*^2^ for the second-order kinetics fit the data accurately, which implied that the D–A reaction between PSF and Ma followed second-order kinetics. The *E*_a,D–A_ and the *k*_0,D–A_ were determined using data obtained at temperatures of 40 °C, 50 °C, 60 °C, and 70 °C, as shown in Figure S4. The calculated *E*_a,D–A_ was found to be 55.35 kJ mol^−1^ (second-order kinetics), which closely aligns with the results obtained by FTIR.

### 3.2. Diels–Alder Reaction between PSF and Mb

**Scheme 3:**
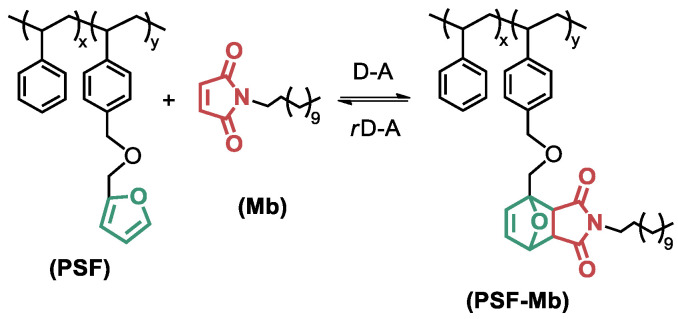
Reversible Diels–Alder reaction between PSF and Mb.

**Figure 11:**
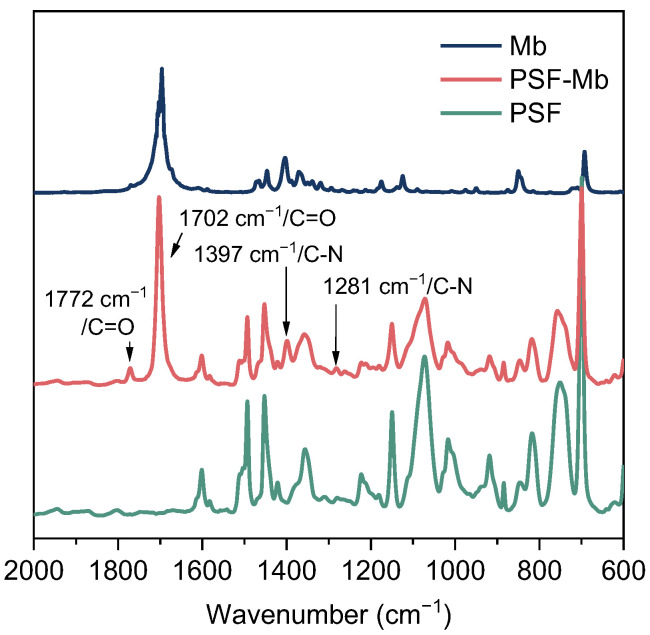
FTIR spectra of PSF, Mb, and PSF-Mb.

Scheme 3 illustrates the reversible D–A reaction between PSF and Mb. The resulting D–A cycloaddition product, PSF-Mb, was identified by FTIR. Figure 11 presents a comparison of the FTIR spectra of PSF, Mb, and PSF-Mb. The emergence or intensification of the characteristic absorption peaks at 1772, 1702, 1397, and 1281 cm^−1^ provide clear evidence of the PSF-Mb adduct.

**Figure 12:**
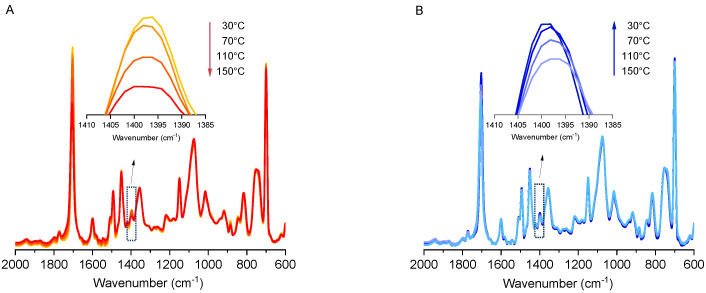
FTIR spectra of (**A**) the heating procedure of PSF-Mb from 30 °C to 150 °C and (**B**) the cooling procedure of PSF-Mb from 150 °C to 30 °C.

Figure 12A shows a gradual decrease in the intensity of the C-N vibration at 1397 cm^−1^ as the temperature of the PSF-Mb sample was increased from 30 °C to 150 °C, whereas a corresponding increase in the intensity of this peak was observed upon cooling the sample from 150 °C to 30 °C, as shown in Figure 12B. These observations suggest that monitoring the absorption at 1397 cm^−1^ enables the tracking of the D–A/*r*D–A reaction in PSF-Mb. Similar to the PSF/Ma system, the PSF/Mb D–A cycloaddition product was subjected to heating at 150 °C, and infrared spectra were collected every 2 min to observe changes in the sample structure over time; subsequently, the PSF-Mb sample was cooled to 70 °C, and spectra were collected every 2 min, as shown in Figure S5.

**Figure 13:**
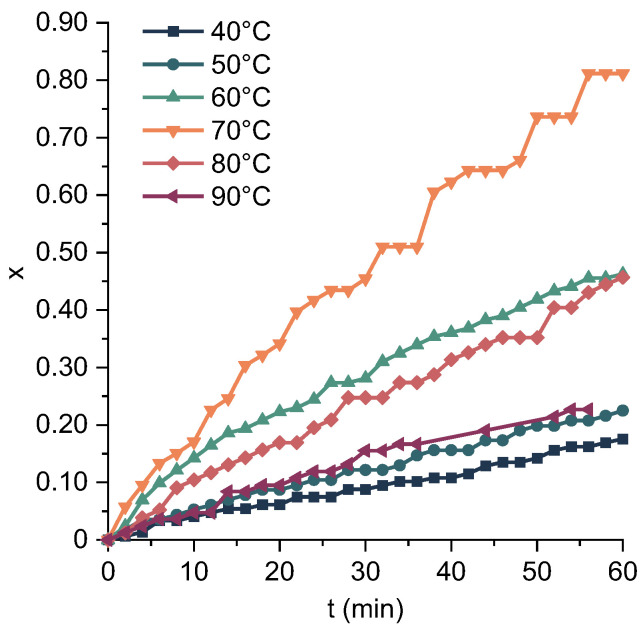
Dependence of conversion x on reaction time in PSF/Mb system derived from FTIR results at 40 °C, 50 °C, 60 °C, 70 °C, 80 °C, and 90 °C, respectively.

The D–A reaction between PSF and Mb was conducted at temperatures of 40 °C, 50 °C, 60 °C, 70 °C, 80 °C, and 90 °C. The absorbance was recorded over time, and the resulting data are shown in Figure S6. By employing Equation (5), the relationship between x and time was calculated at various temperatures during the D–A reaction and is plotted in Figure 13. The data reveal that the slope of the curves decreased as the temperature exceeded 80 °C, similar to the trend observed in the PSF/Ma system.

The apparent kinetic coefficient *k*_app_ of the D–A reaction in PSF-Mb was determined from Figure S7 based on Equations (2)–(4), and the resulting *k*_1_, *k*_2_, and *k*_3_ values are presented in Table 2. Based on the *R*^2^ values, both first-order kinetics and second-order kinetics were found to fit the entire temperature range. Figure S8 depicts the relationship between *k* and temperature. The *E*_a,D–A_ was calculated using Equation (6) based on the data obtained at 40 °C, 50 °C, 60 °C, and 70 °C, and the resulting values of *E*_a,D–A_ were found to be approximately 60~80 kJ mol^−1^, as shown in Table 2. Notably, the second-order kinetics results fit the data well.

**Figure 14:**
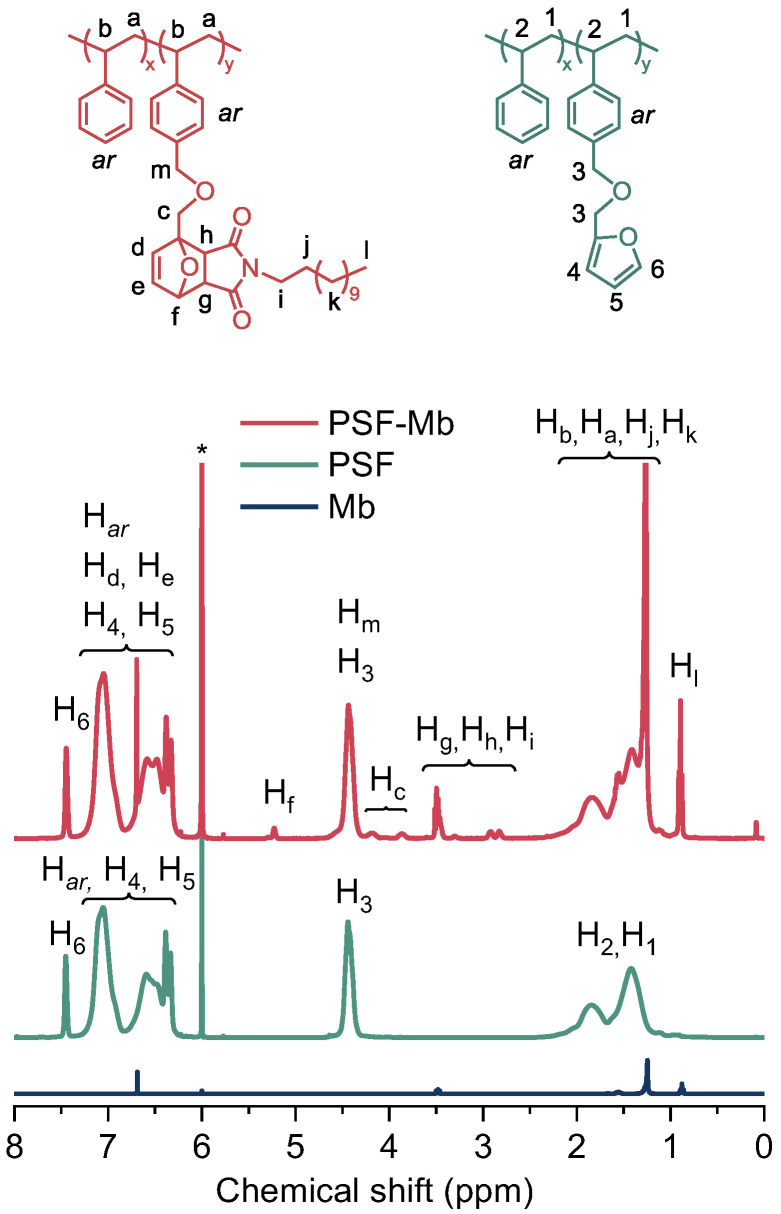
^1^H-NMR spectra of PSF, Mb, and PSF-Mb. (* solvent: C_2_D_2_Cl_4_).

PSF-Mb was characterized using ^1^H-NMR spectroscopy, as presented in Figure 14. In comparison with PSF, PSF-Mb displayed distinct D–A peaks at chemical shifts of 5.15~5.35 ppm, 3.75~4.28 ppm, 3.55~3.65 ppm, 3.20~3.36 ppm, and 2.70~3.05 ppm. The composition of PSF-Mb can be determined by the characteristic peak integral at 3.75~4.28 ppm in conjunction with the integral at 3.75~4.75 ppm. The results indicate that 19.7 mol% of the furyl group in PSF underwent the D–A reaction, and the F/M ratio (molar ratio of furan and maleimide) in PSF-Mb was found to be 5:1.

**Figure 15:**
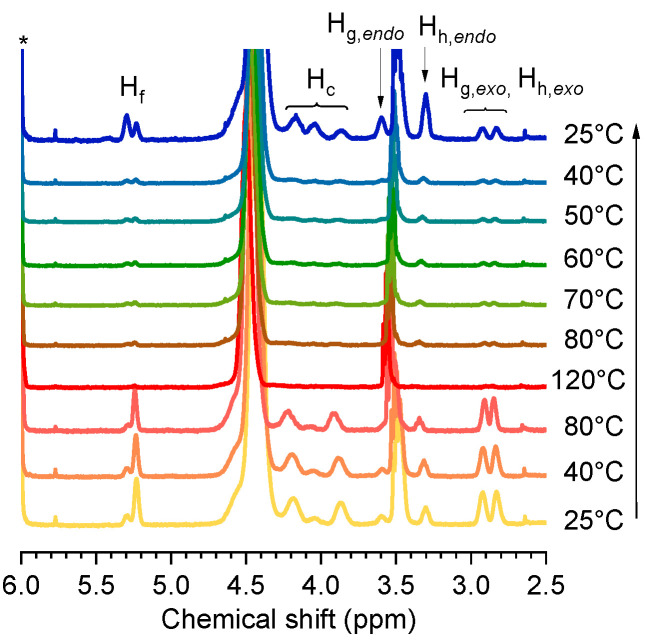
^1^H-NMR spectra of PSF-Mb at variable temperatures. (* solvent: C_2_D_2_Cl_4_).

**Figure 16:**
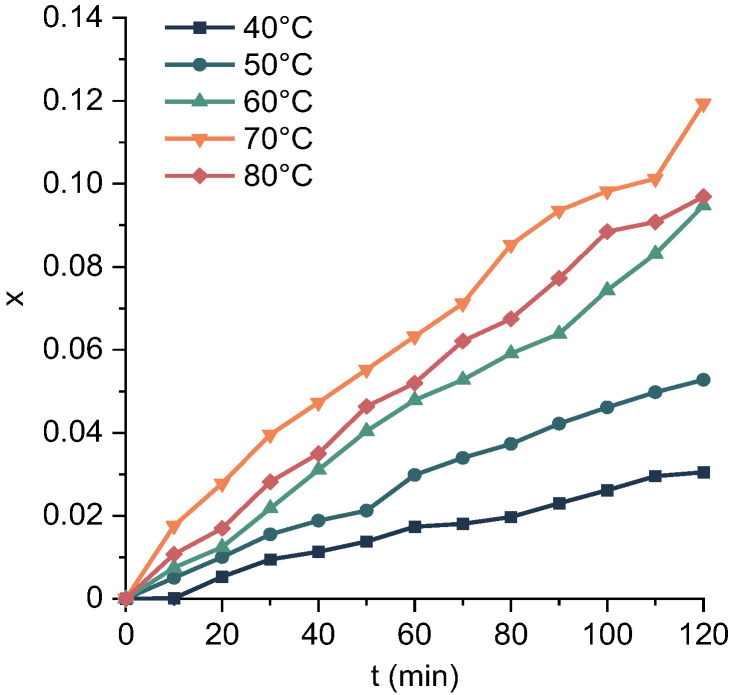
Dependence of conversion x on reaction time in PSF/Mb system derived from ^1^H-NMR results at 40 °C, 50 °C, 60 °C, 70 °C, and 80 °C, respectively.

The reaction between PSF and Mb was conducted in C_2_D_2_Cl_4_ under nitrogen, and the reaction progress was monitored in real time using ^1^H-NMR spectroscopy. A temperature cycle ranging from 25 °C to 120 °C was employed to obtain a variable-temperature ^1^H-NMR spectrum, as depicted in Figure 15. To investigate the kinetics of the D–A reaction, the PSF-Mb adduct was subjected to the *r*D–A reaction at 120 °C and subsequently cooled to various temperatures (40 °C, 50 °C, 60 °C, 70 °C, and 80 °C) for 2 h in C_2_D_2_Cl_4_, as shown in Figure S9. The real-time ^1^H-NMR spectrum was acquired every 10 min at each designed temperature, following the measurement method utilized in the PSF/Ma system. The results showed that the characteristic peak of the D–A adduct disappeared completely after 20 min at 120 °C, indicating the occurrence of the *r*D–A reaction at high temperature. The D–A reaction conversion x was calculated based on Equation (7), and the relationship between x and time at each temperature is depicted in Figure 16.

The kinetics of the D–A reaction were monitored using ^1^H-NMR, as shown in Figure S10. The *k*_1_, *k*_2_, and *k*_3_ values were calculated and are listed in Table 2. The values of *k*_1_, *k*_2_, and *k*_3_ increased as the temperature rose from 40 °C to 70 °C, but decreased at 80 °C, indicating that 40~70 °C is optimal for promoting the rapid completion of the D–A reaction. Additionally, the second-order kinetics fit the data accurately, as evidenced by the linear fitting value *R*^2^, indicating that the D–A reaction between PSF and Mb may follow second-order kinetics. By analyzing the data obtained at temperatures of 40 °C, 50 °C, 60 °C, and 70 °C, the *E*_a,D–A_ and *k*_0,D–A_ for the reaction were determined, as shown in Figure S11. The calculated *E*_a,D–A_ (second-order) was 41.53 kJ mol^−1^.

By comparing the experimental results obtained by ^1^H-NMR and FTIR at 70 °C, it was observed that the value of *k*_2_ for the PSF/Mb system in the bulk state (determined by FTIR) was 3.09 × 10^−2^ min^−1^·mol^−1^·L, whereas that in the solution state (determined by ^1^H-NMR) was 1.04 × 10^−3^ min^−1^·mol^−1^·L. This represents a difference of an order of magnitude between the two systems. Moreover, the *E*_a,D–A_ values of the two systems were significantly different: 69.59 kJ mol^−1^ for the bulk system and 41.53 kJ mol^−1^ for the solution system. This is in contrast to the experimental results obtained for the PSF/Ma system. We propose that the reason for this difference is that both PSF and Ma molecules contain phenyl, which results in their structural similarity, enabling good miscibility and mutual melting between the two reactants, thus facilitating mutual diffusion and reaction in solution or in bulk state. This similarity leads to the similar calculated *k* and *E*_a,D–A_ in the two states. However, in the PSF/MB system, the Mb molecule contains a long alkyl substituent, which is less compatible with the PSF polymer than the Ma molecule. This may result in the local aggregation of Mb after the *r*D–A reaction between PSF and Mb in the bulk state, resulting in a higher local concentration and a faster rate of the D–A reaction in the bulk state than in solution.

## 4. Conclusions

In this work, polystyrene containing furan substituents PSF was synthesized via the copolymerization of styrene and furan-functionalized styrene, and the PSF was further reacted with two different structurally designed maleimide small molecules, Ma and Mb, in a D–A reaction to produce PSF-Ma and PSF-Mb adducts, respectively. The reversible D–A reaction kinetics of the PSF/Ma and PSF/Mb systems in the bulk state were investigated using infrared spectroscopy, and the reaction kinetic parameters, including the apparent kinetic coefficient *k*_app_, the Arrhenius activation energy *E*_a,D–A_, and pre-exponential factor *k*_0,D–A_, were calculated. The D–A reaction kinetics of these two systems were also studied using ^1^H-NMR in solution, and the results were compared with those from infrared spectroscopy. The results showed that the infrared spectroscopy provided reliable data for studying the D–A reaction kinetics in the PSF/Ma system, as the *E*_a,D–A_ and *k*_app_ values obtained from the infrared spectra in the bulk state were comparable to those obtained from the ^1^H-NMR spectra in solution. However, in the PSF/Mb system, the *E*_a,D–A_ value obtained from the infrared spectra was significantly higher than that obtained from the ^1^H-NMR spectra, while the *k*_app_ value showed a similar trend. The poor compatibility between aliphatic Mb and aromatic PSF in the bulk polymer matrix caused the heterogeneous environment, which is considered to be the main reason for the inconsistency between the results obtained from the bulk- and solution-state experiments. These results inspired us to consider the compatibility between substituents on furan and maleimide moieties when designing D–A reactants, as it might directly affect the efficiency of the D–A reaction and even the final properties of the materials.

## Figures and Tables

**Table 1:** Kinetics parameters of the Diels–Alder reaction between PSF and Ma.

Kinetic Order	Method	*k*_app_ (*k*_1_/min^−1^, *k*_2_/min^−1^·mol^−1^·L, *k*_3_/min^−1^·mol^−2^·L^2^)					*k*_0, D–A_ /min^−1^·mol^1−n^·L^n−1^	*E*_a, D–A_ /kJ mol^−1^
		40 °C	50 °C	60 °C	70 °C	80 °C		
n = 1	FTIR	5.44 × 10^−4^ (*R*^2^ = 0.918)	8.02 × 10^−4^ (*R*^2^ = 0.983)	1.13 × 10^−3^ (*R*^2^ = 0.981)	3.08 × 10^−3^ (*R*^2^ = 0.971)	2.50 × 10^−3^ (*R*^2^ = 0.990)	7.62 × 10^4^	49.17 (*R*^2^ = 0.915)
	^1^H-NMR	5.21 × 10^−4^ (*R*^2^ = 0.995)	7.70 × 10^−4^ (*R*^2^ = 0.994)	1.41 × 10^−3^ (*R*^2^ = 0.995)	2.86 × 10^−3^ (*R*^2^ = 0.996)	2.20 × 10^−3^ (*R*^2^ = 0.994)	1.43 × 10^5^	50.84 (*R*^2^ = 0.977)
n = 2	FTIR	5.50 × 10^−4^ (*R*^2^ = 0.918)	8.20 × 10^−4^ (*R*^2^ = 0.984)	1.18 × 10^−3^ (*R*^2^ = 0.982)	3.38 × 10^−3^ (*R*^2^ = 0.976)	2.73 × 10^−3^ (*R*^2^ = 0.992)	1.89 × 10^5^	51.54 (*R*^2^ = 0.914)
	^1^H-NMR	5.38 × 10^−4^ (*R*^2^ = 0.995)	8.05 × 10^−4^ (*R*^2^ = 0.994)	1.54 × 10^−3^ (*R*^2^ = 0.995)	3.45 × 10^−3^ (*R*^2^ = 0.998)	2.53 × 10^−3^ (*R*^2^ = 0.995)	8.16 × 10^5^	55.35 (*R*^2^ = 0.971)
n = 3	FTIR	1.11 × 10^−3^ (*R*^2^ = 0.919)	1.68 × 10^−3^ (*R*^2^ = 0.985)	2.45 × 10^−3^ (*R*^2^ = 0.983)	7.43 × 10^−3^ (*R*^2^ = 0.981)	5.97 × 10^−3^ (*R*^2^ = 0.992)	9.46 × 10^5^	53.92 (*R*^2^ = 0.911)
	^1^H-NMR	1.11 × 10^−3^ (*R*^2^ = 0.994)	1.69 × 10^−3^ (*R*^2^ = 0.994)	3.35 × 10^−3^ (*R*^2^ = 0.993)	8.37 × 10^−3^ (*R*^2^ = 0.995)	5.82 × 10^−3^ (*R*^2^ = 0.992)	9.67 × 10^6^	59.96 (*R*^2^ = 0.964)

**Table 2:** Kinetics parameters of the Diels–Alder reaction between PSF and Mb.

Kinetic Order	Method	*k*_app_ (*k*_1_/min^−1^, *k*_2_/min^−1^·mol^−1^·L, *k*_3_/min^−1^·mol^−2^·L^2^)					*k*_0, D–A_ /min^−1^·mol^1−n^·L^n−1^	*E*_a, D–A_ /kJ mol^−1^
		40 °C	50 °C	60 °C	70 °C	80 °C		
n = 1	FTIR	3.01 × 10^−3^ (*R*^2^ = 0.989)	4.01 × 10^−3^ (*R*^2^ = 0.994)	1.00 × 10^−2^ (*R*^2^ = 0.993)	2.16 × 10^−2^ (*R*^2^ = 0.991)	9.64 × 10^−3^ (*R*^2^ = 0.989)	3.35 × 10^7^	60.68 (*R*^2^ = 0.954)
	^1^H-NMR	2.65 × 10^−4^ 0.987	4.60 × 10^−4^ 0.995	8.00 × 10^−4^ 0.993	9.78 × 10^−4^ 0.990	8.66 × 10^−4^ 0.996	1.37 × 10^3^	40.11 (*R*^2^ = 0.973)
n = 2	FTIR	3.32 × 10^−3^ (*R*^2^ = 0.987)	4.59 × 10^−3^ (*R*^2^ = 0.993)	1.42 × 10^−2^ (*R*^2^ = 0.996)	3.09 × 10^−2^ (*R*^2^ = 0.979)	1.31 × 10^−2^ (*R*^2^ = 0.971)	1.11 × 10^9^	69.59 (*R*^2^ = 0.955)
	^1^H-NMR	2.69 × 10^−4^ (*R*^2^ = 0.988)	4.73 × 10^−4^ (*R*^2^ = 0.996)	8.39 × 10^−4^ (*R*^2^ = 0.993)	1.04 × 10^−3^ (*R*^2^ = 0.991)	9.13 × 10^−4^ (*R*^2^ = 0.997)	2.40 × 10^3^	41.53 (*R*^2^ = 0.975)
n = 3	FTIR	7.35 × 10^−3^ (*R*^2^ = 0.983)	1.05 × 10^−2^ (*R*^2^ = 0.989)	4.08 × 10^−2^ (*R*^2^ = 0.984)	9.07 × 10^−2^ (*R*^2^ = 0.949)	3.61 × 10^−2^ (*R*^2^ = 0.939)	9.62 × 10^10^	79.18 (*R*^2^ = 0.952)
	^1^H-NMR	5.47×10^−4^ (*R*^2^ = 0.988)	9.73×10^−4^ (*R*^2^ = 0.996)	1.76×10^−3^ (*R*^2^ = 0.991)	2.23×10^−3^ (*R*^2^ = 0.991)	1.93×10^−3^ (*R*^2^ = 0.996)	8.95×10^3^	43.12 (*R*^2^ = 0.978)

## Data Availability

Data are contained within the article and Supplementary Materials.

## Supplementary Materials

The following supporting information can be downloaded at: https://www.mdpi.com/article/10.3390/polym16030441/s1, Figure S1: ^1^H-NMR spectra of monomers St and *f*St, homopolymers PS and PF, and copolymer PSF. Figure S2: The FTIR absorbance during the D–A reaction between PSF and Ma recorded with time at different temperatures: 40 °C, 50 °C, 60 °C, 70 °C, 80 °C and 90 °C. Figure S3: The ^1^H-NMR spectra during the D–A reaction between PSF and Ma recorded with time at different temperatures: 40 °C, 50 °C, 60 °C, 70 °C and 80 °C. Figure S4: Fitting the Arrhenius plot to (A) first-order, (B) second-order, and (C) third-order reaction to determine the *E*_a,D–A_ and *k*_0,D–A_ of the D–A reaction between PSF and Ma based on the ^1^H-NMR results. Figure S5: FTIR spectra of (A) holding the temperature at 70 °C for 60 min and (B) holding the temperature at 150 °C for 20min. Figure S6: The FTIR absorbance during the D–A reaction between PSF and Mb recorded with time at different temperatures: 40 °C, 50 °C, 60 °C, 70 °C, 80 °C and 90 °C. Figure S7: The FTIR absorbance during the D–A reaction between PSF and Mb recorded with time at different temperatures: 40 °C, 50 °C, 60 °C, 70 °C, 80 °C and 90 °C. Figure S8: Fitting the Arrhenius plot to (A) first-order, (B) second-order, and (C) third-order reaction to determine the *E*_a,D–A_ and *k*_0,D–A_ of the D–A reaction between PSF and Mb based on the FTIR results. Figure S9: The ^1^H-NMR spectra during the D–A reaction between PSF and Mb recorded with time at different temperatures: 40 °C, 50 °C, 60 °C, 70 °C and 80 °C. Figure S10: Fitting the conversion data to (A) first-order, (B) second-order, and (C) third-order reaction kinetics to determine the apparent kinetic coefficient *k*_app_ of D–A reaction between PSF and Mb at 40 °C, 50 °C, 60 °C, 70 °C and 80 °C based on the ^1^H-NMR results. Figure S11: Fitting the Arrhenius plot to (A) first-order, (B) second-order, and (C) third-order reaction to determine the *E*_a,D–A_ and *k*_0,D–A_ of the D–A reaction between PSF and Mb based on the ^1^H-NMR results.
