# Supplementary material for: Kinetic Study of the Diels–Alder Reaction between Maleimide and Furan-Containing Polystyrene Using Infrared Spectroscopy
Source: Polymers (Basel). 2024 Feb 5;16(3):441. doi: 10.3390/polym16030441 (PMC10857387; doi:10.3390/polym16030441)
Supplement: Supplementary file 1 [file polymers-16-00441-s001.zip › polymers-2788965-supplementary.pdf]

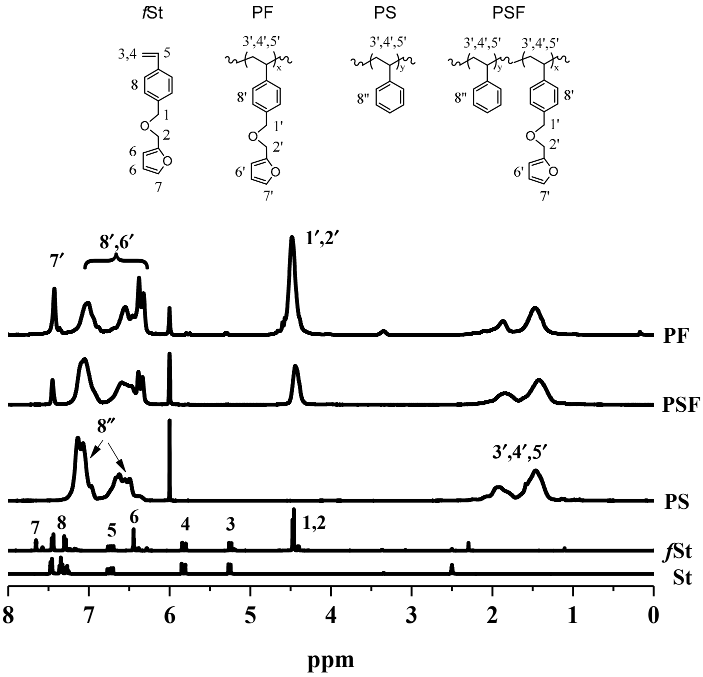

Figure S1 ^{1}H-NMR spectra of monomers St and *f*St, homopolymers PS and PF, and copolymer PSF

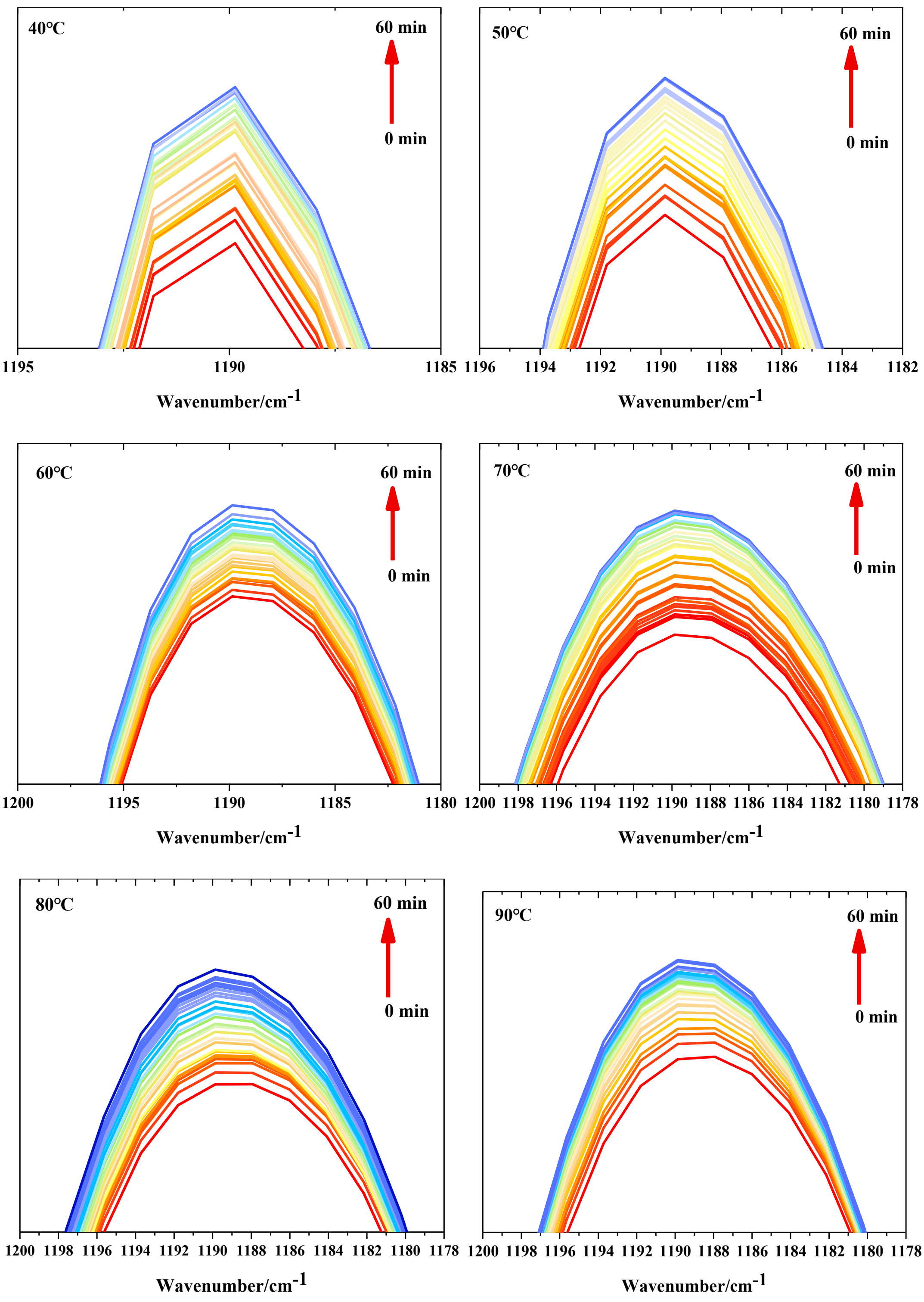

Figure S2 The FTIR absorbance during the D-A reaction between PSF and Ma recorded with time at different temperatures: 40°C, 50°C, 60°C, 70°C, 80°C and 90°C

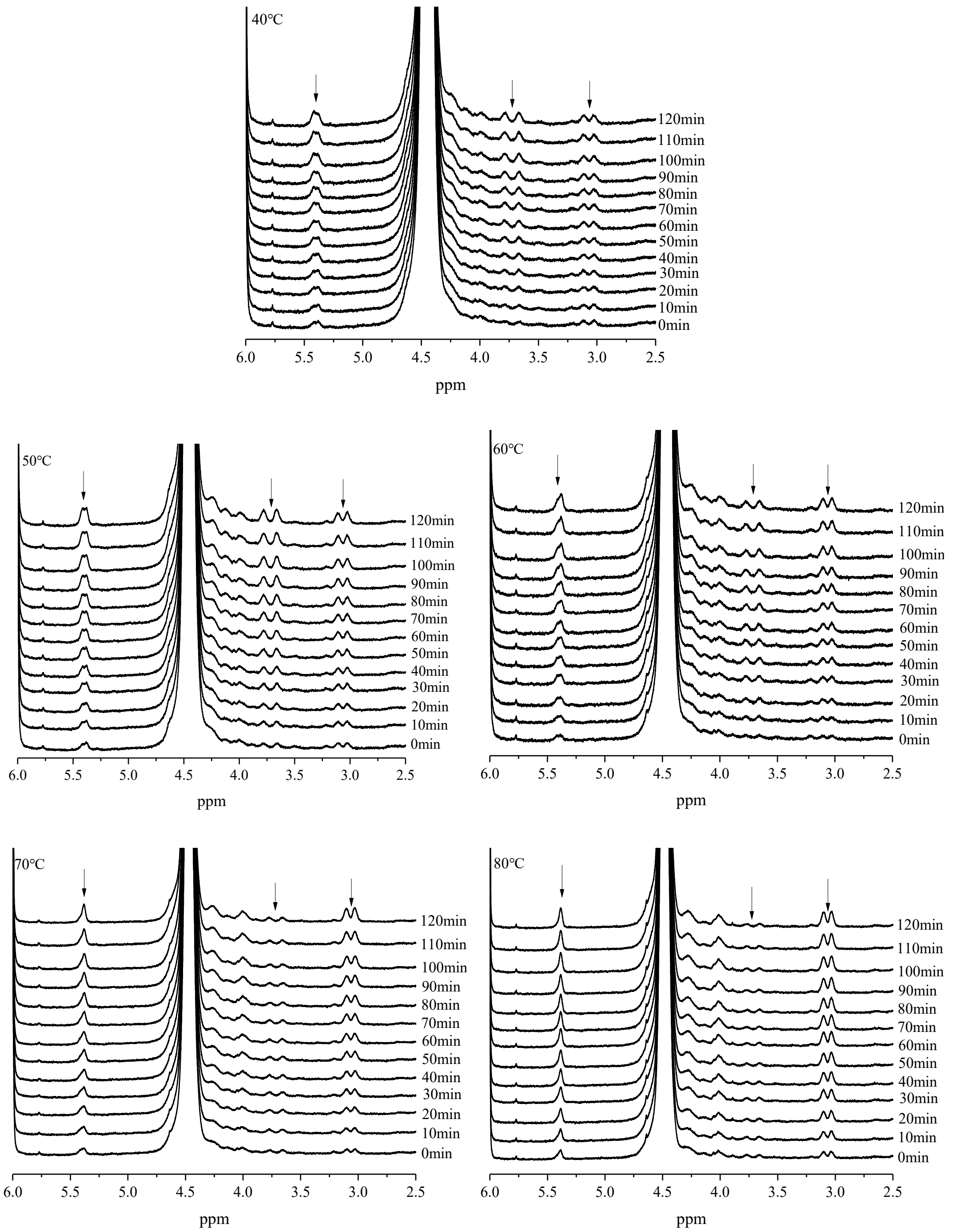

Figure S3 The ^{1}H-NMR spectra during the D-A reaction between PSF and Ma recorded with time at different temperatures: 40°C, 50°C, 60°C, 70°C and 80°C

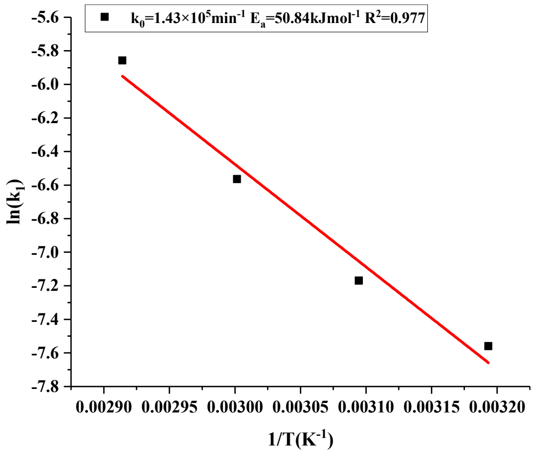

(A)

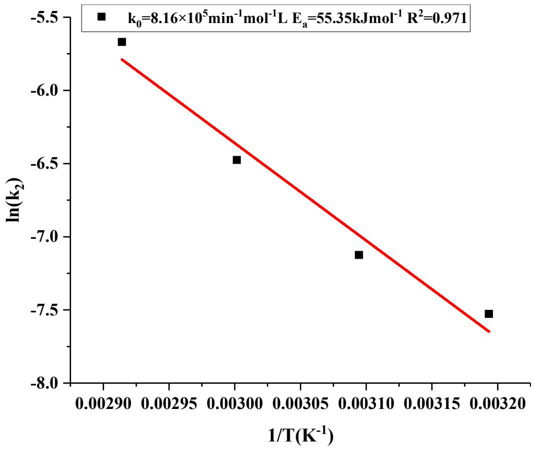

(B)

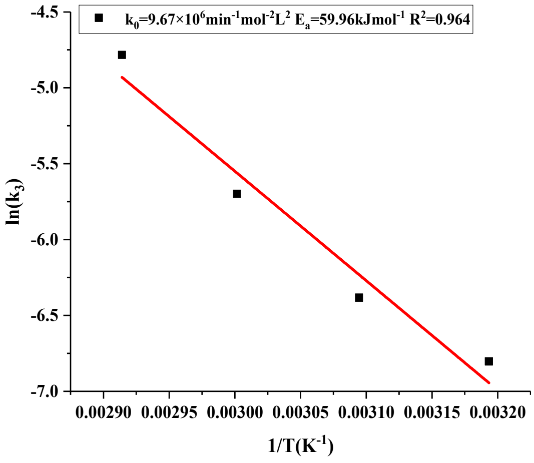

(C)

Figure S4 Fitting the Arrhenius plot to (A) first-order, (B) second-order, and (C) third-order reaction to determine the $E_{a,D\text{-}A}$ and $k_{0,D\text{-}A}$ of the D-A reaction between PSF and Ma based on the ^{1}H-NMR results

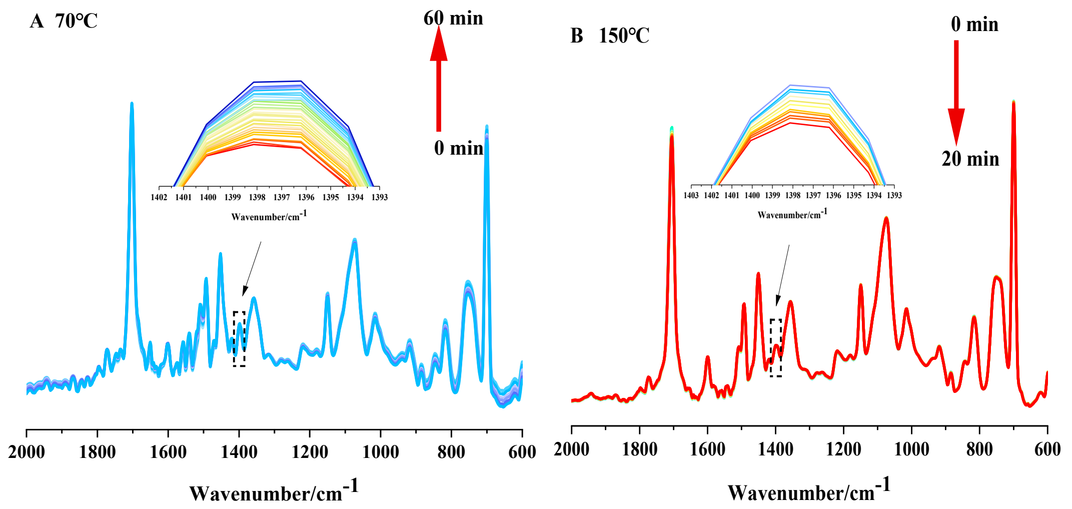

Figure S5 FTIR spectra of (A) holding the temperature at 70°C for 60 min and (B) holding the temperature at 150°C for 20min

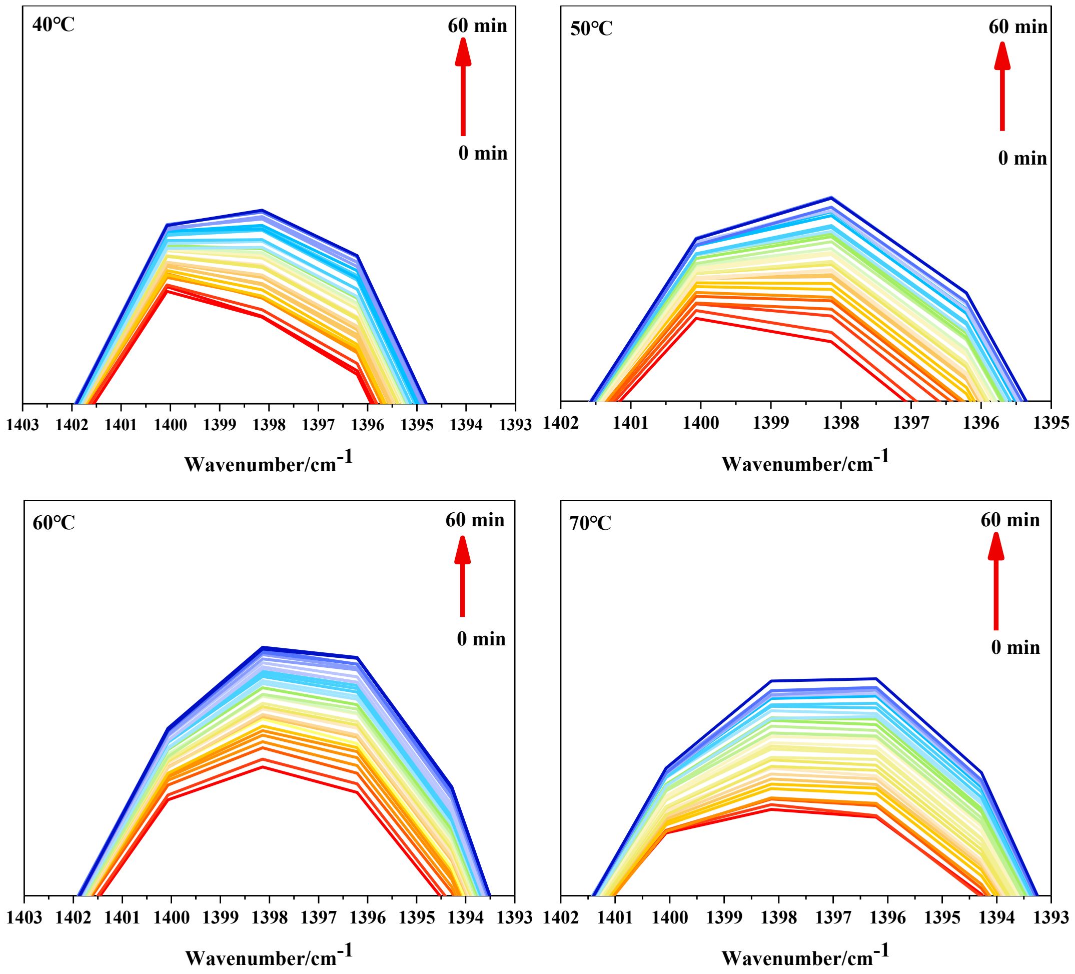

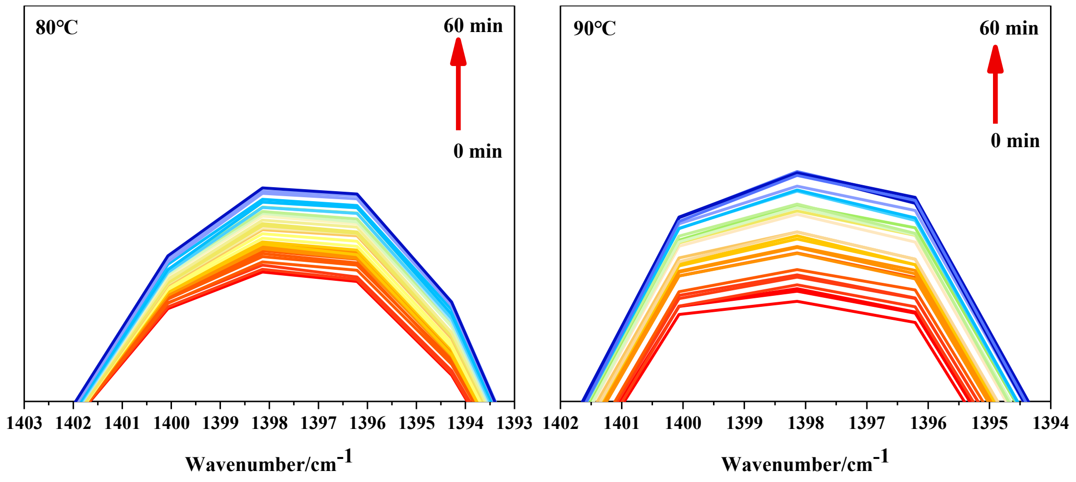

Figure S6 The FTIR absorbance during the D-A reaction between PSF and Mb recorded with time at different temperatures: 40°C, 50°C, 60°C, 70°C, 80°C and 90°C

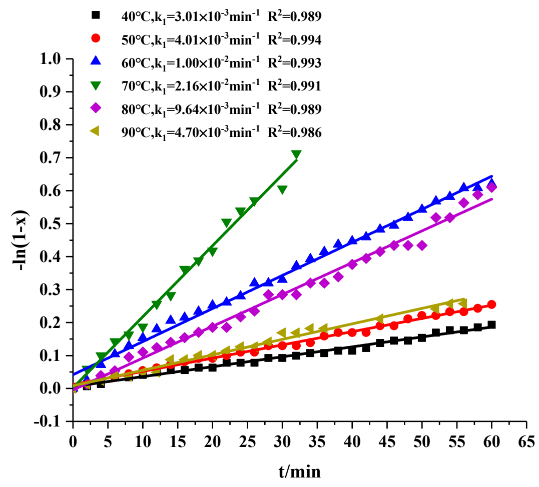

(A)

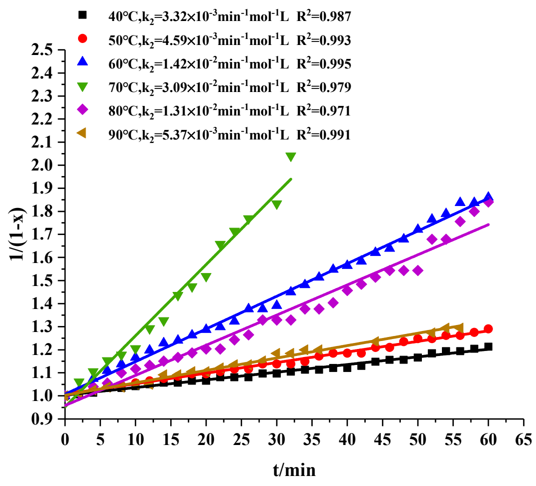

(B)

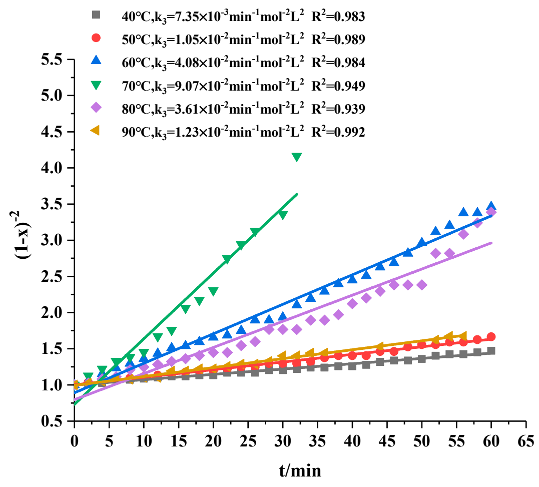

(C)

Figure S7 Fitting the conversion data to (A) first-order, (B) second-order, and (C) third-order reaction kinetics to determine the apparent kinetic coefficient k_{app} of D-A reaction between PSF and Mb at 40°C, 50°C, 60°C, 70°C, 80°C and 90°C based on the FTIR results

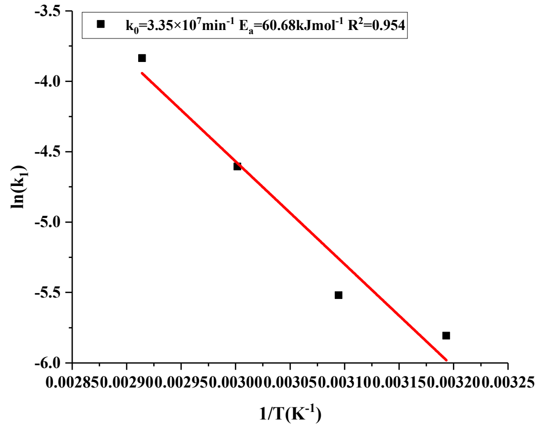

(A)

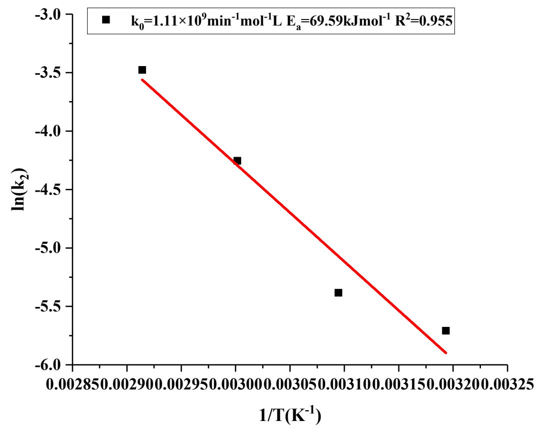

(B)

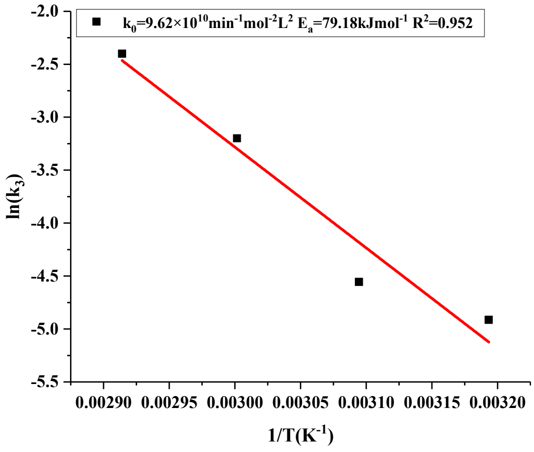

(C)

Figure S8 Fitting the Arrhenius plot to (A) first-order, (B) second-order, and (C) third-order reaction to determine the $E_{a,\text{D-A}}$ and $k_{0,\text{D-A}}$ of the D-A reaction between PSF and Mb based on the FTIR results

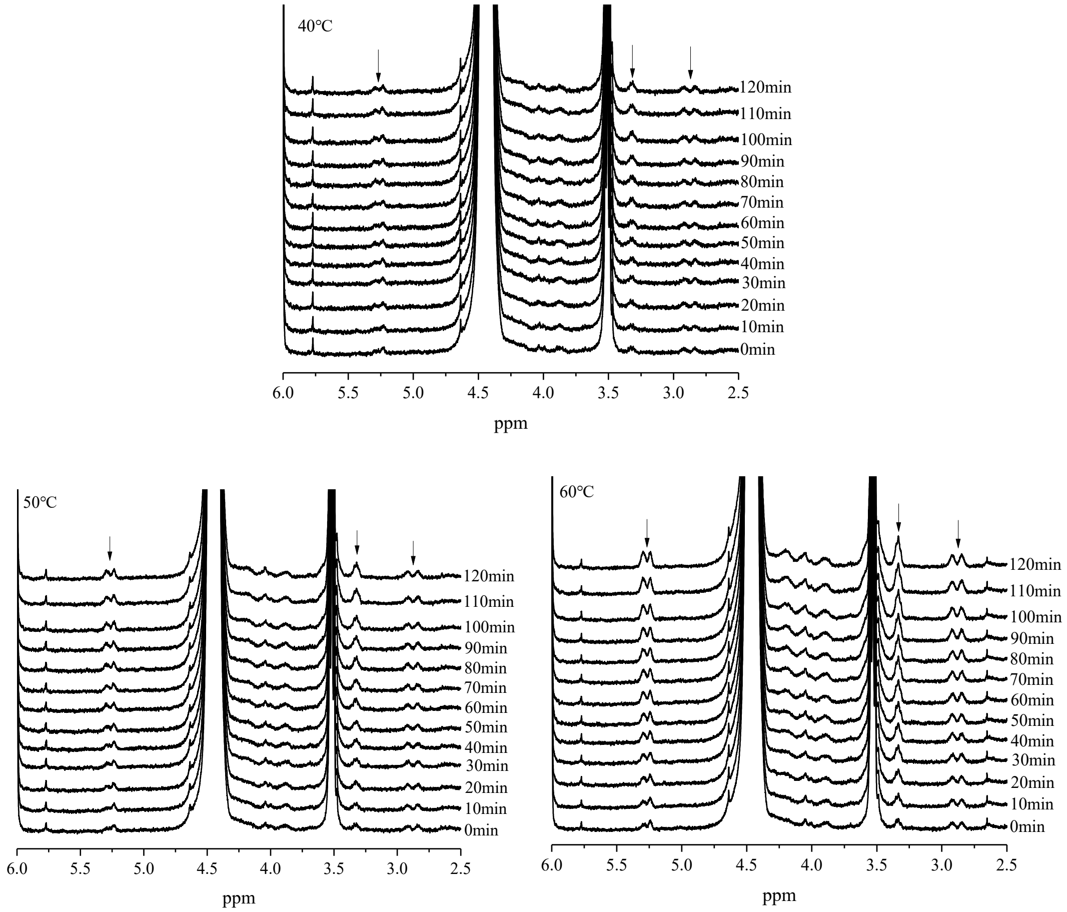

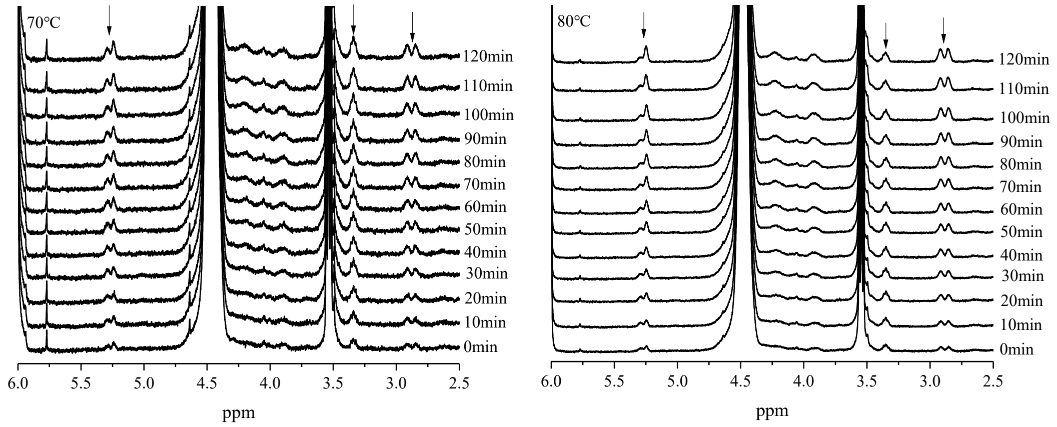

Figure S9 The ^{1}H-NMR spectra during the D-A reaction between PSF and Mb recorded with time at different temperatures: 40°C, 50°C, 60°C, 70°C and 80°C

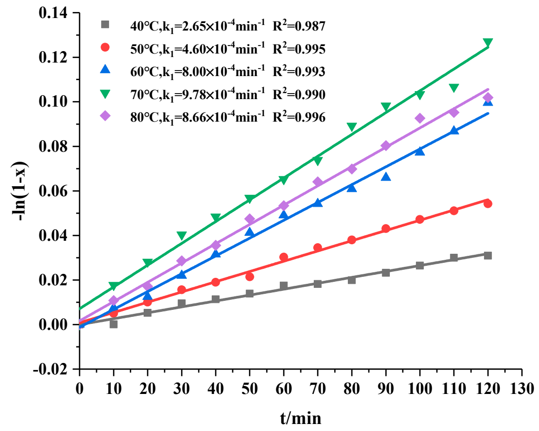

(A)

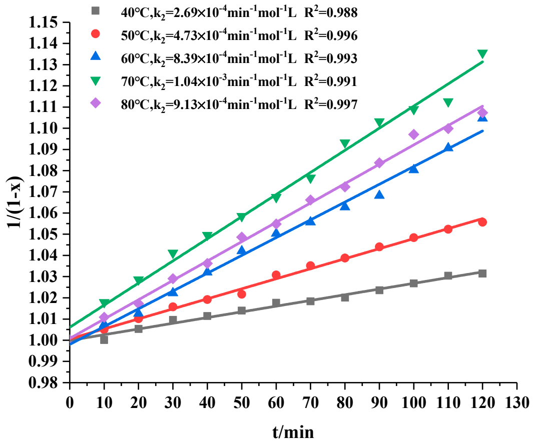

(B)

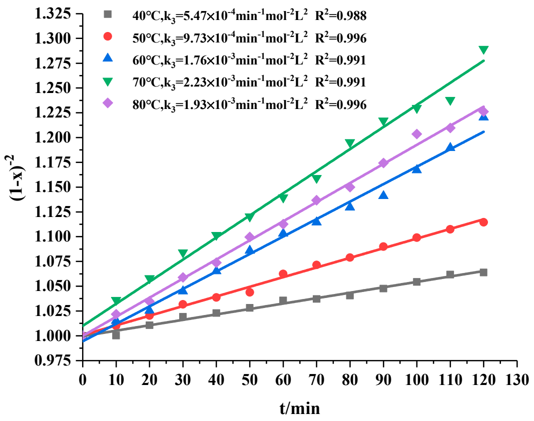

(C)

Figure S10 Fitting the conversion data to (A) first-order, (B) second-order, and (C) third-order reaction kinetics to determine the apparent kinetic coefficient k_{app} of D-A reaction between PSF and Mb at 40°C, 50°C, 60°C, 70°C and 80°C based on the ^{1}H-NMR results

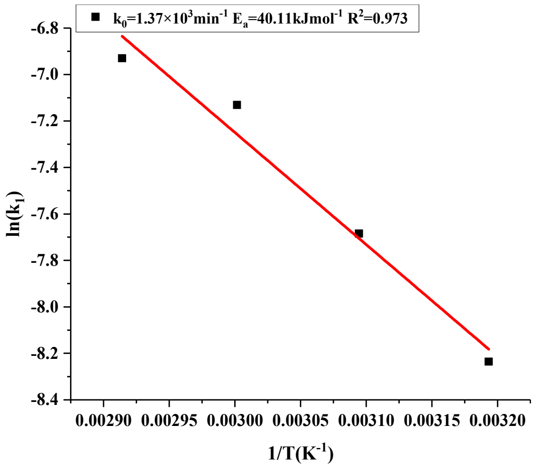

(A)

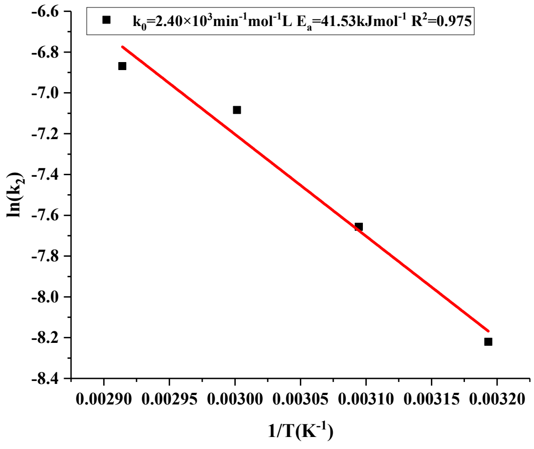

(B)

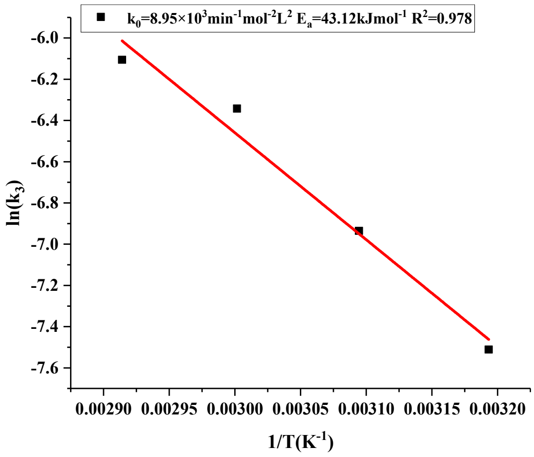

(C)

Figure S11 Fitting the Arrhenius plot to (A) first-order, (B) second-order, and (C) third-order reaction to determine the $E_{a,D\text{-}A}$ and $k_{0,D\text{-}A}$ of the D-A reaction between PSF and Mb based on the ^{1}H-NMR results
